# The use of gene interaction networks to improve the identification of cancer driver genes

**DOI:** 10.7717/peerj.2568

**Published:** 2017-01-26

**Authors:** Emilie Ramsahai, Kheston Walkins, Vrijesh Tripathi, Melford John

**Affiliations:** 1 Department of Mathematics & Statistics, The Faculty of Science and Technology, The University of the West Indies, St. Augustine Campus, Trinidad and Tobago; 2 Department of Preclinical Sciences, The University of the West Indies, St. Augustine, Trinidad and Tobago

**Keywords:** Driver genes, Interaction network, Algorithm, Gene expression, Mutation, Weighted network, Cancer, Graph

## Abstract

Bioinformaticians have implemented different strategies to distinguish cancer driver genes from passenger genes. One of the more recent advances uses a pathway-oriented approach. Methods that employ this strategy are highly dependent on the quality and size of the pathway interaction network employed, and require a powerful statistical environment for analyses. A number of genomic libraries are available in R. DriverNet and DawnRank employ pathway-based methods that use gene interaction graphs in matrix form. We investigated the benefit of combining data from 3 different sources on the prediction outcome of cancer driver genes by DriverNet and DawnRank. An enriched dataset was derived comprising 13,862 genes with 372,250 interactions, which increased its accuracy by 17% and 28%, respectively, compared to their original networks. The study identified 33 new candidate driver genes. Our study highlights the potential of combining networks and weighting edges to provide greater accuracy in the identification of cancer driver genes.

## Introduction

Cellular signaling pathways are composed of a number of proteins between which information is transmitted via chemical reactions. This flow of signals between cells and within cells allows them to respond appropriately to biological needs. Such processes form extremely complex and carefully regulated pathways that branch out to reach a number of effector proteins. As a consequence of this, a single protein is able to influence multiple cellular processes such as cell division, protein synthesis, and cell death. Each component may modify signals it receives before passing them on to downstream targets. Interactions include protein–protein binding, protein degradation, phosphorylation, and protein–DNA binding. Intracellular pathways do not operate in isolation, but are cross-linked to other pathways that together form a huge web.

Cancer is characterized by uncontrolled cell proliferation. It develops when genetic aberrations disrupt a number of signaling processes that promote the bypassing of normal restrictions that keep cell proliferation in check. An understanding of mutated genes that drive the formation of cancer is important in the discovery of new drugs and the recommendation of targeted treatment regimes for patients.

**Figure 1:**
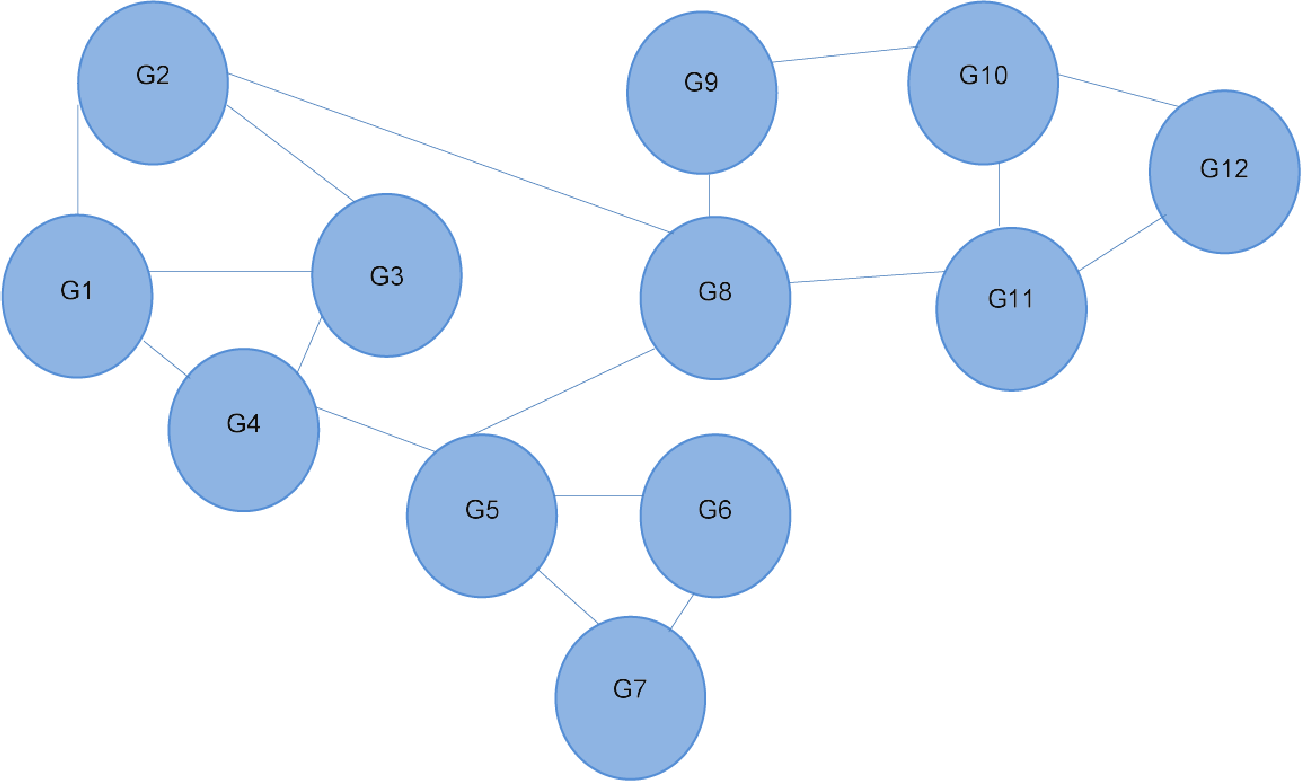
Interaction network of 12 different genes. Each line depicts an interaction between two genes. G4 is shown to interact directly with three other genes, and indirectly with all the others. Referred to as the 12-node network.

**Figure 2:**
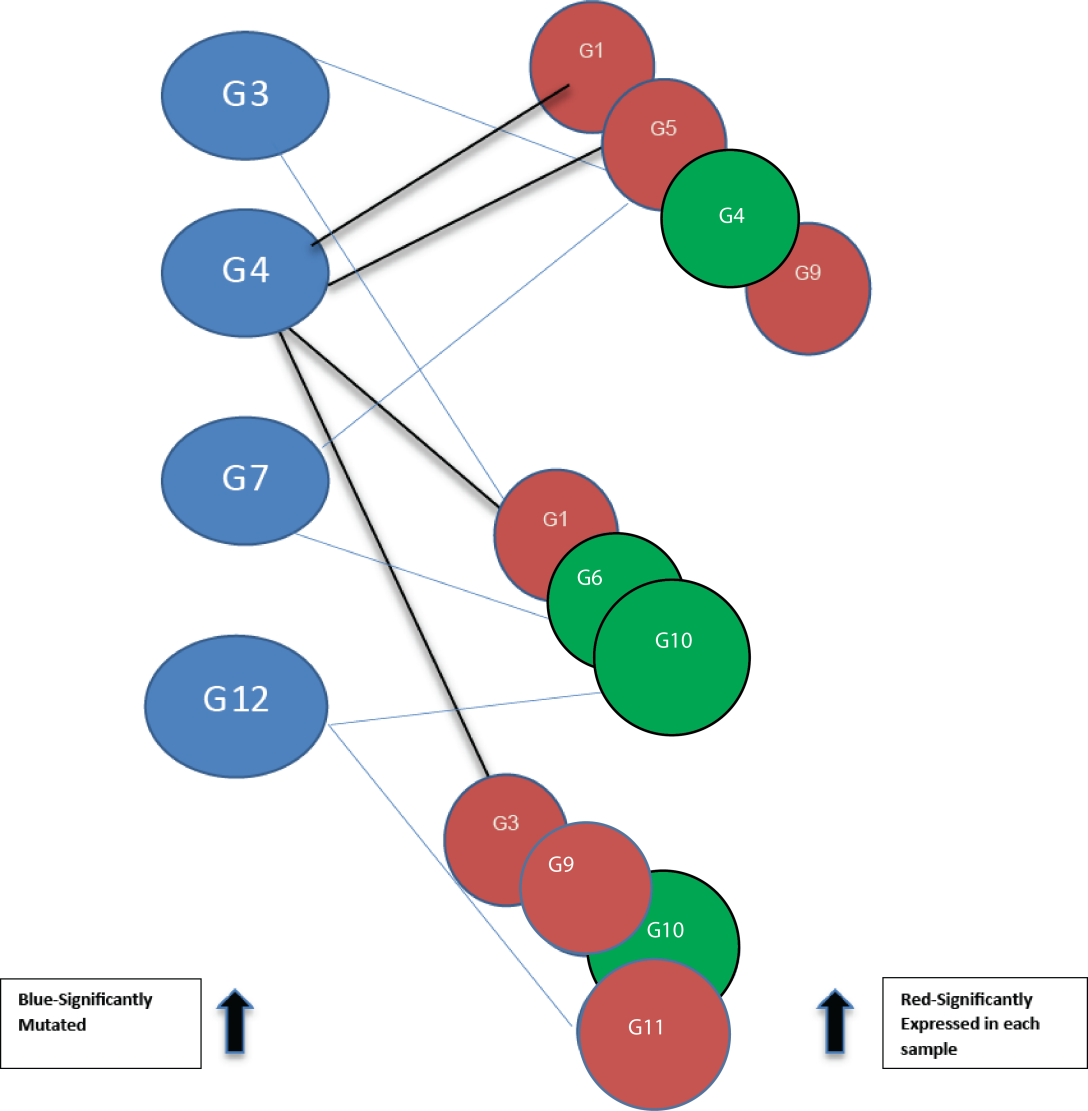
Bipartite graph constructed from the 12-node network in Fig. 1. Blue nodes represent mutated genes. Those in red represent significantly expressed genes in three different samples while the green nodes are not significantly expressed.

Pathway databases are constructed from data obtained from publications by the scientific community. They range in size and scope from mathematical models such as BioModels (Chelliah & Laibe, 2013) to much larger, community-curated reaction databases such as Reactome (Croft et al., 2014); the National Cancer Institute Pathway Information Database (PID) (Schaefer et al., 2009); and, the Kyoto Encyclopedia of Genes and Genomes (KEGG) (Kanehisa et al., 2012). Whilst a number of commercial pathway knowledge bases are available for performing traditional pathway analysis (Khatri, Sirota & Butte, 2012), factors such as cost, data format, sharing restrictions, and terms of use impose limitations that make them less attractive as sources of data for network analysis. At present, all biological pathway databases are incomplete. Database consolidation has been challenging (Fearnley et al., 2014), but possible by the adoption of the Proteomics Standards Initiative—Molecular Interaction (PSI-MI) format, and the more simplified tabular format, MITAB (Kerrien et al., 2007). Different network modeling techniques applied on experimental data in predicting interactions (Kumar & Ranganathan, 2013) have also contributed in producing repositories of large-scale pathway reaction and interaction data. In the development of pathway-based tools and methods, these interaction networks are often represented as graphs for analysis. As a result, the number of interaction networks in graph format is growing, and integration is considered at the graph level.

In this paper we seek to determine if combining interaction graphs improves the identification of cancer driver genes by DriverNet and DawnRank. They were both developed using the R environment, which provides powerful data analysis and graphical features. DriverNet met the standards set by Bioconductor (Gentleman et al., 2004). We combined graphs from DriverNet (Bashashati et al., 2012), VarWalker (Jia & Zhao, 2014), and DawnRank (Hou & Ma, 2014) for our analyses.

### DriverNet

DriverNet uses a greedy algorithm to identify driver genes from a bipartite graph combining mutation frequency and differential expression. It utilizes the protein functional interaction network constructed by Wu, Feng & Stein (2010), which was constructed from various sources of information such as curated pathways with non-curated data including protein–protein interactions, gene co-expression, protein domain interaction, gene ontology (GO) annotations, and text-mined protein interactions. These provide various small molecules, proteins, complexes, post-translationally modified proteins, and nucleic acid sequences which are mapped onto the genome using online repositories such as UniProt (2010) and Entrez Genes (Maglott et al., 2011). It determines which interactions form part of the network by using a Bayes classifier, and eliminates those that do not fit. DriverNet predicts driver genes by considering the effect of mutated genes on the gene expression levels of interacting partners.

Using threshold cut-off values genes are categorized as expressed or not. In Fig. 2, blue nodes partition of the bipartite graph represent mutated genes whilst nodes in red represent their expression status for different patients. Genes in red are significantly expressed. The gene interaction network connects nodes between the two sets. In the identification of candidate driver genes, the guiding principle is to select as many red nodes as possible using the fewest number of blue nodes. At each stage of the greedy algorithm, mutated genes with the highest number of significant connections (such as G4 in Fig. 2) are selected as candidate driver genes.

### VarWalker

VarWalker uses a Random Walk with Restart (RWR) algorithm. The network it uses was constructed using the Human Protein Reference Database (Keshava Prasad et al., 2009), a manually curated resource. It includes protein–protein interactions, catalytic reactions, and protein translocation events that have been evaluated against other repositories of human protein–protein interaction data in the public domain (Mathivanan et al., 2006). This network shows the Cancer Genome Census (CGC) genes (Futreal et al., 2004) tend to be located more closely to each other than other genes. Specifically, 71% of CGC genes are directly connected and 26% have a shortest path of two. VarWalker uses this trait to nominate candidate driver genes by ascertaining consensus across multiple samples for mutated genes that converge. An initial gene filtering process removes long genes that are more frequently mutated due to size.

**Figure 3:**
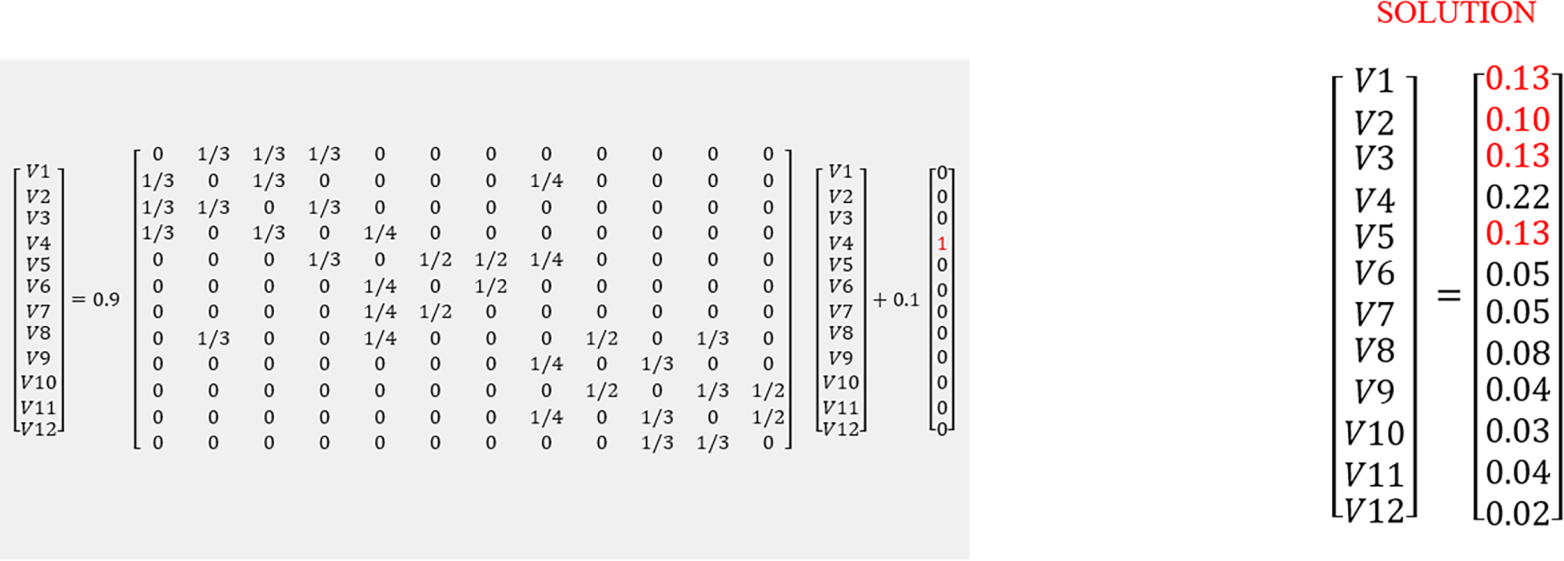
Matrix illustration of random walk with restart. In Eq. (1) V is the proximity vector (V1…V12), *r* is the restart probability of 0.1. Matrix A represents the network. *P* is the vector where the 4th element is 1, as the walker is at G4 at time 0. The solution shows nearby nodes with higher scores. The red values (0.13 and 0.10) were determined to be closer to G4.

The RWR method calculates a vector V that represents the proximity between a given node and all other nodes in the network by solving Eq. (1) in Fig. 3. The gene network is represented by a matrix A (see Fig. 3), if a gene i links to a gene j, i.e. i–j, then Aij<>0. In this case, its value would be the probability of moving to node j from i. If gene i does not link to gene j, then Aij = 0. In Fig. 1, from G4, it is possible to move directly to one of the three other nodes. The probability of moving to a directly connected node is proportional to the number of outgoing nodes from G4, in this case 1/3. The RWR is applicable as a proximity metric because after a sufficiently long time interval, the probability of being at G4 at a random time provides a measure of the proximity between G4 and all the other nodes. Figure 3 is the matrix representation of this equation for the 12-gene network in Fig. 1.

(1) \begin{eqnarray*}\mathrm{V }=(1-r)\mathrm{AV }+r\mathrm{P}.\end{eqnarray*}

In this example a restart probability value of 0.1 is used for *r*. The 12 by 12 matrix A presented in Fig. 3 is derived from the 12-node network in Fig. 1. P is the vector in which the ith element holds the probability that the walker is at node i at time 0. In this case we start at G4, so the fourth element of P is 1, and all others are zero (see Fig. 3). The value of V is then calculated, to satisfy Eq. (1). The solution of this equation is vector V given in Fig. 3:

\begin{eqnarray*}\mathrm{V }=(0.13,0.10,0.13,0.22,0.13,0.05,0.05,0.08,0.04,0.03,0.04,0.02). \end{eqnarray*}

This solution indicates nearby nodes (G1, G3, and G5) with higher scores of 0.13. We can also determine G9 and G11 are equally distant from G4. With a large network, this can be computationally intensive, thus, this matrix solution can be replaced by an iterative solution.

### DawnRank

DawnRank selects potential driver genes based on their impact on the overall differential expression of its downstream genes in the interaction network. In this network, all redundant edges are collapsed to single edges when aggregating networks from different databases. With this method an individual patient sample is used rather than a large cohort, so drivers are identified on a personalized level. This single patient approach is totally independent of the mutation frequency, and can therefore be considered focused on finding more infrequent or rare drivers. It classifies rare and even patient-specific mutations. This is the use of the ‘long tail phenomenon’ when selecting driver genes, which considers cancer mutations as being characterized by a small number of frequently mutated genes and a large number of infrequently mutated genes. Selected genes are compared to CGC and Pan Cancer standard driver gene list (Cancer Genome Atlas Research et al., 2013; Tamborero et al., 2013) for validation.

**Figure 4:**
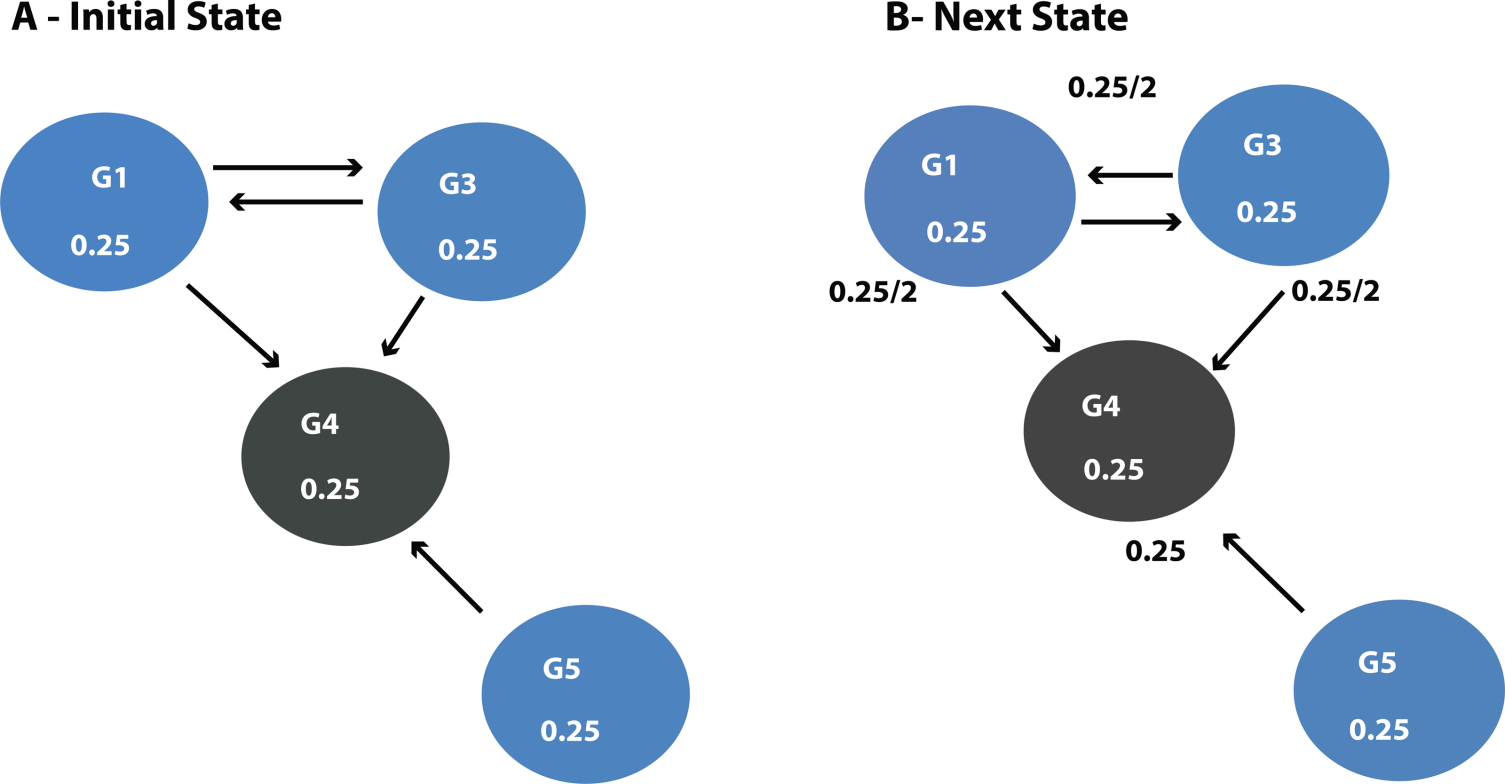
(A) Initial state *t* = 0, a rank of 1 is divided equally between all 4 nodes. To get to (B) next state *t* = 1, ranks are updated by adding up ranks of all incoming genes divided by the number of outgoing links from each of them.

**Figure 5:**
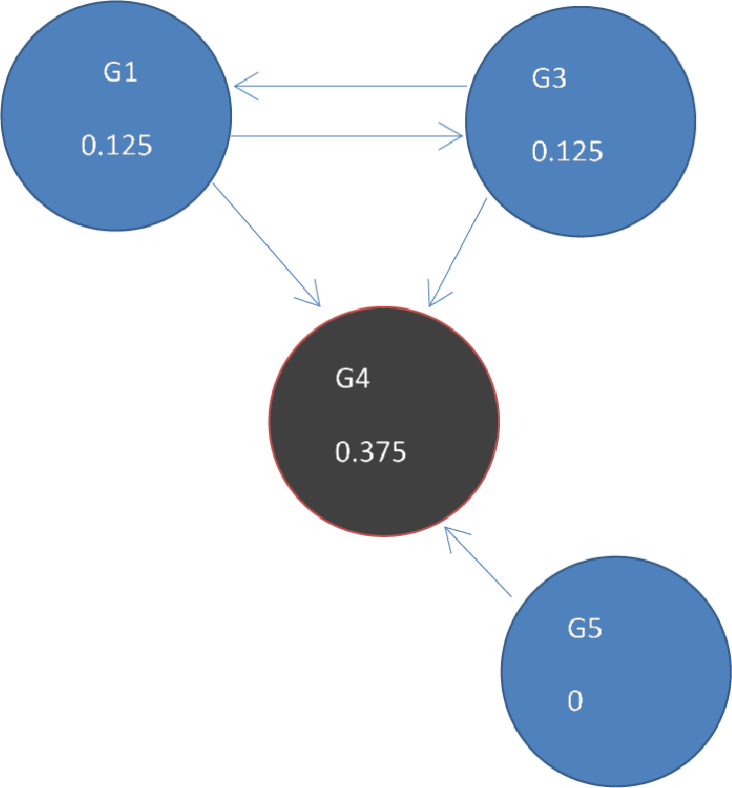
Page Rank results at state *t* = 1 after one iteration where ranks are recalculated as outlined in Fig. 4.

DawnRank uses the PageRank family of algorithms to rank genes based on incoming links. PageRank (Page et al., 1999) was developed to measure the human interest in web pages. It is used by Google’s search engine to rank web pages. To illustrate this, consider a sub-network from our 12-node network in Fig. 1 as shown in Fig. 4A. Each of the 4 genes G1, G3, G4, and G5 is initially given the same rank. The initial rank for each of the 4 genes is therefore 0.25. Fig. 4A illustrates the topology of this simplified network at initial state *t* = 0. The next iteration involves updating the rank of each gene by adding up the rank of each incoming gene divided by the number of outgoing links from it. This is illustrated in Fig. 4B. Thus, new ranks shown in Fig 5 are calculated as follows:

\begin{eqnarray*}\begin{array}{@{}llll@{}} \displaystyle \mathrm{G1}=\mathrm{G3}/2=0.25/2=0.125;\mathrm{G3}=\mathrm{G1}/2=0.25/2=0.125;&\displaystyle &\displaystyle &\displaystyle \text{}\\ \displaystyle \mathrm{G4}=\mathrm{G1}/2+\mathrm{G3}/2+\mathrm{G3}/1=0.25/2+0.25/2+0.25/1=0.375;\mathrm{G5}=0.&\displaystyle &\displaystyle &\displaystyle \text{} \end{array} \end{eqnarray*}

The ranks of genes may be weighted so that a gene is given a higher rank, even though there are fewer links to it, if more important genes link to it. The output of the PageRank algorithm is a list of genes and their rankings based on the gene network configuration. A high PageRank score for a mutated gene in cancer indicates that the gene is more likely to be a driver.

For DawnRank’s implementation of the PageRank algorithm the initial rank value for each gene would be 1/11,648, as the network of genes consists of 11,648 members. A gene linked to many other genes with high ranks receives a high rank. This process is modeled using states, the transitions from one state to another depending only on the current state rather than a preceding state. This is the Markov property, where each iteration is equally probable. The difference in the ranks between time *t* = 0 and *t* = 1 is computed recursively as *r*_*t*+1_ and *r*_*t*_, until it converges to an insignificant value (epsilon). It can also stop after a set number of iterations, which is 100 for DawnRank.

## Methods

### Interaction network construction

**Figure 6:**
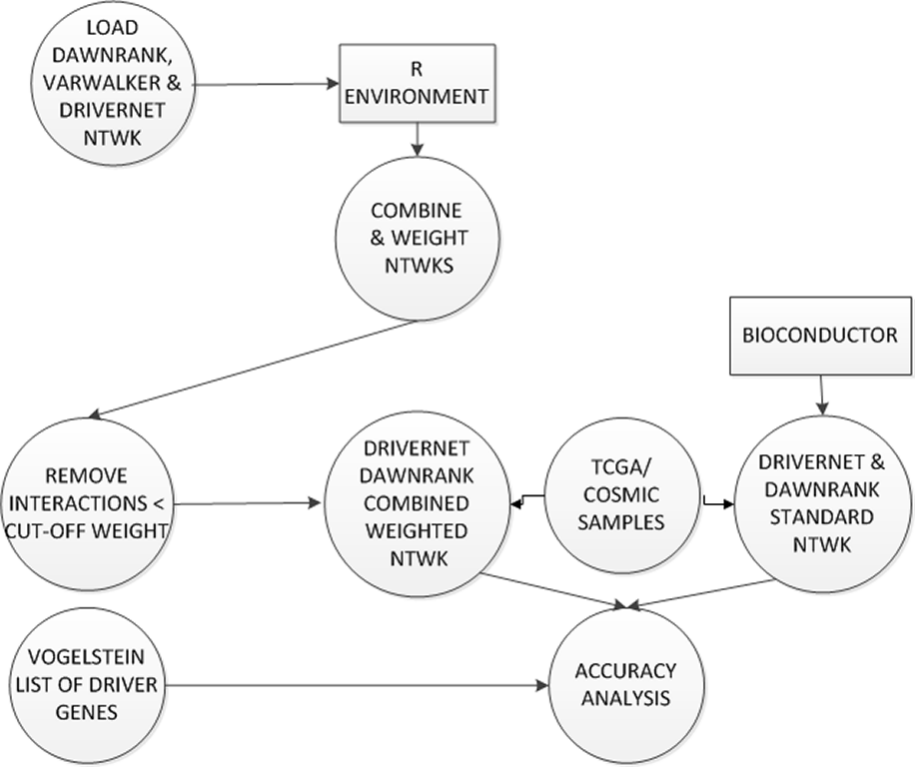
Construction and testing of weighted combined network. Each interaction in the combined network was weighted. Low scoring interactions were removed. This new weighted network was used by DriverNet and DawnRank in the prediction of driver genes. These were analysed against the published list of driver genes from Vogelstein et al. (2013).

Network data files from the DriverNet, DawnRank and VarWalker packages were used. These were transformed into individual graphs and combined using the igraph library (Csardi & Nepusz, 2006) as shown in the dataflow diagram in Fig. 6.

**Figure 7:**
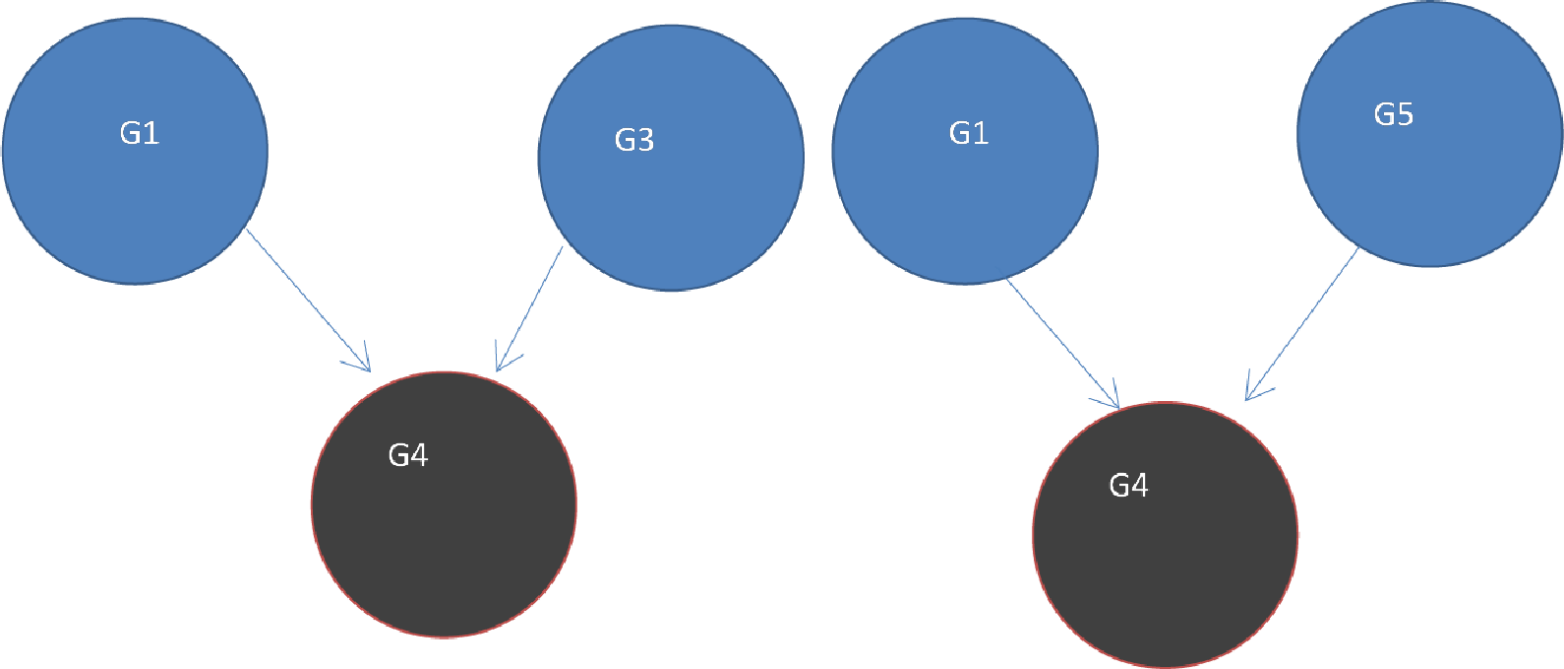
Two V-structures. A V-structure configuration exists in the network based on the paths among a group of any 3 genes. Two of the genes directly linked into the third. The edge G1->G4 forms part of both of these V-structures G1->G4<-G3 and G1->G4<-G5.

For each of the three individual networks and the combined network we computed an interaction score assuming independence. To infer these interaction scores, we combined for each interaction between gene G_i_ and gene G_j_, two scores:

• Q_ij_ is the number of graphs the interaction between gene G_i_ and gene G_j_ occurs in, represented on a common scale [0, 1]. This is where q_ij_ = 0 represents no information about the interaction, and q_ij_ = 1 represents strong evidence for the interaction as it occurs in all the graphs;
• R_ij_ is a count of the number of v-structures the edge G_i_->G_j_ is part of in the network projected onto a common scale [0, 1] (see Fig. 7).

Q_ij_ and R_ij_ were represented as matrices, with genes identifying both rows and columns, q_ij_ and r_ij_ are the scores for the interaction between gene G_i_ and gene G_j_. These two were combined as S_ij_ as in Eq. (2). This schema for combining scores allows us to adjust *w* between [0, 1] depending on the confidence in each of these contributors to the final weighting. For our implementation we used equal weighting by setting *w* to 0.5

(2) \begin{eqnarray*}{\mathrm{S}}_{\mathrm{ij}}={w\mathrm{Q}}_{\mathrm{ij}}+(1-w){\mathrm{R}}_{\mathrm{ij}}.\end{eqnarray*}

### Assessing the linear bias correction

In order to quantify the impact of the dependency on the interaction scores, we compared the sum of the interactions from the individual graphs to those produced from the combined network for those interactions common in all three networks. The individual scores were summed as in Eq. (3) across the 3 graphs, and projected onto a common scale [0, 1].

(3) \begin{eqnarray*}\mathrm{Sum}=\sum {\mathrm{S}}_{\mathrm{i}}.\end{eqnarray*}

Eq. (4) was used to calculate the bias. We applied a linear regression between the summed values and the calculated values using Eq. (2) for the combined network.

(4) \begin{eqnarray*}\text{Combined network}=\alpha \ast \mathrm{sum}+\beta .\end{eqnarray*}

The parameters *α* and *β* represent the bias for the interaction weights of the combined network.

### Testing the weighted network

Interactions with a weight less than or equal to the cut-off value of 0.17 were discarded. The resultant network was used to analyse the prediction of driver genes by DriverNet and DawnRank.

Data consisting of 504 samples of breast cancer (BRCA) initially from TCGA were derived from DawnRank. These included somatic mutation and differential gene expression data between the cancer and normal transcriptome. Driver genes were predicted by DawnRank using its standard network and the weighted combined network. Mutation and expression datasets consisting of 178 cervical cancer samples were downloaded from the Catalogue of Somatic Mutation in Cancer (COSMIC) (Forbes et al., 2011). These were transformed into two binary matrices where the rows were patients and the columns were genes. For the purpose of our analysis, expression values between the range −2 and 2 were considered to be normal. Thus in the expression matrix, if a *z*-score value was >2.0 or <−2 the binary matrix element was set to 1 (TRUE), otherwise it was set to 0 (FALSE). Glioblastoma Multiforme (GBM) samples from The Cancer Genome Atlas (TCGA) (Cancer Genome Atlas Research, 2008) were used from DriverNet. These were represented by 2 matrices with 200 rows and 1,255 columns. Driver genes were predicted using DriverNet for GBM and cervical cancer.

The analysis included sensitivity, specificity, accuracy and receiver operating characteristic (ROC) with Area under the curve (AUC) measures (Zhu, Zeng & Wang, 2010).

Genes predicted as candidate driver genes were classified as true positives if they were present in the 125 driver genes from Vogelstein et al. (2013). Details on this analysis can be found in Supplementary Files.

## Results and Discussion

The methods employed by the packages DriverNet, VarWalker and DawnRank to predict cancer driver genes involve the use of well-established algorithms that are very different. There are also significant differences in the pathway data sources used for the construction of their interaction networks, and in their use of gene mutation and expression data (see Tables 1 and 2). All packages use mutation data of tumor samples, but only DawnRank and DriverNet use gene expression data. Whereas DawnRank uses mainly expression data, DriverNet uses a combination of both. One would therefore expect there to be wide variations in their prediction of cancer driver genes. Our analysis shows this to be the case, when looking at the accuracy measures in Table 3.

**Table 1:** Characteristics of packages used to predict cancer driver genes.

	DawnRank	VarWalker	DriverNet
Base algorithm	PageRank	Random Walk with Restart	Greedy optimisation on bipartite graph
Data type	Expression mainly	Mutation only	Mutation and expression
Data source	TCGA	TCGA	TCGA
Reference list	CGC, Pan Cancer	CGC	CGC

**Table 2:** Network characteristics of packages used to predict cancer driver genes.

	DawnRank network	VarWalker network	DriverNet network	Weighted combined network
Pathway data source	Reactome, NCI-Nature, Kegg, PDI	Human Protein Reference Database (HPRD)	Reactome, NCI-Nature, Kegg, Panther Pathways, Cell Map, NCI-BioCarta, TRED	Interactions with a weight greater than 0.17
Nodes	11,648	8,768	1,255	13,862
Interactions	211,794	73,182	130,153	372,250
Density	0.00156	0.0009	0.0827	0.00193
Diameter	14	14	6	9

**Table 3:** Comparison of sensitivity, specificity, and accuracy of driver gene prediction using the standard network and the weighted combined network for DriverNet and DawnRank.

	Parameter	Standard network	Weighted combined network
Glioblastoma multiforme (DriverNet)	Sensitivity	0.8274	0.9796
	Specificity	0.5400	0.2393
	**Accuracy**	**0.8159**	**0.9734**
Cervical (DriverNet)	Sensitivity	0.74440	0.91713
	Specificity	0.58000	0.52991
	**Accuracy**	**0.7378**	**0.9139**
Breast (DawnRank)	Sensitivity	0.68284	0.96719
	Specificity	0.61429	0.36752
	**Accuracy**	**0.6796**	**0.9621**

**Figure 8:**
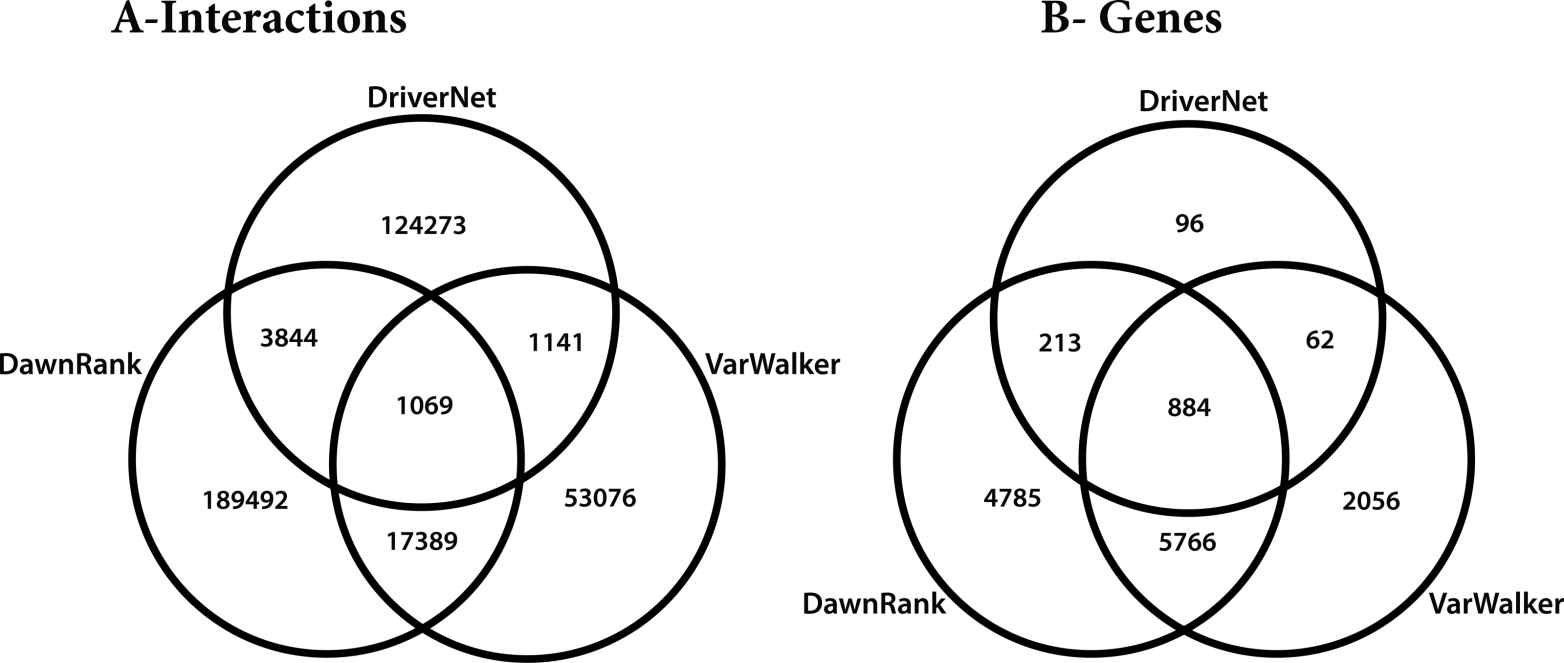
Venn diagram of the 3 networks and how they overlap (A) number of interactions (B) number of genes.

The differences in these networks were highlighted by considering how they overlap. All of the graphs partly overlap, but only 6% of the interactions are reported in more than one of the graphs. We considered the five different subgroups among the 3 graphs (Fig. 8), which included: unique genes and interactions; those reported in DawnRank and Varwalker but not in DriverNet; those reported in Varwalker and DriverNet but not in DawnRank; those reported in DriverNet and DawnRank but not in Varwalker; and also those reported in all three packages.

The Human Protein Reference Database (HPRD) used for the construction of the VarWalker network is not used by either of the other 2 packages (see Table 2). There are differences in the methods employed by DriverNet (Wu, Feng & Stein, 2010) and DawnRank (Ciriello et al., 2012) to determine pairwise interactions though there are some similarities in the pathway data used. Differences in the methods employed lead to significant differences in the number of nodes and interactions (see Table 2).

The use of the weighted combined network made a significant improvement to the prediction of driver genes using Vogelstein’s list as a reference (see Table 3). The accuracy increased from 81% to 97% for GBM and from 73% to 91% for cervical cancer by DriverNet. The largest accuracy increase was reported for breast cancer by DawnRank, a 28% increase from 68% to 96%.

**Figure 9:**
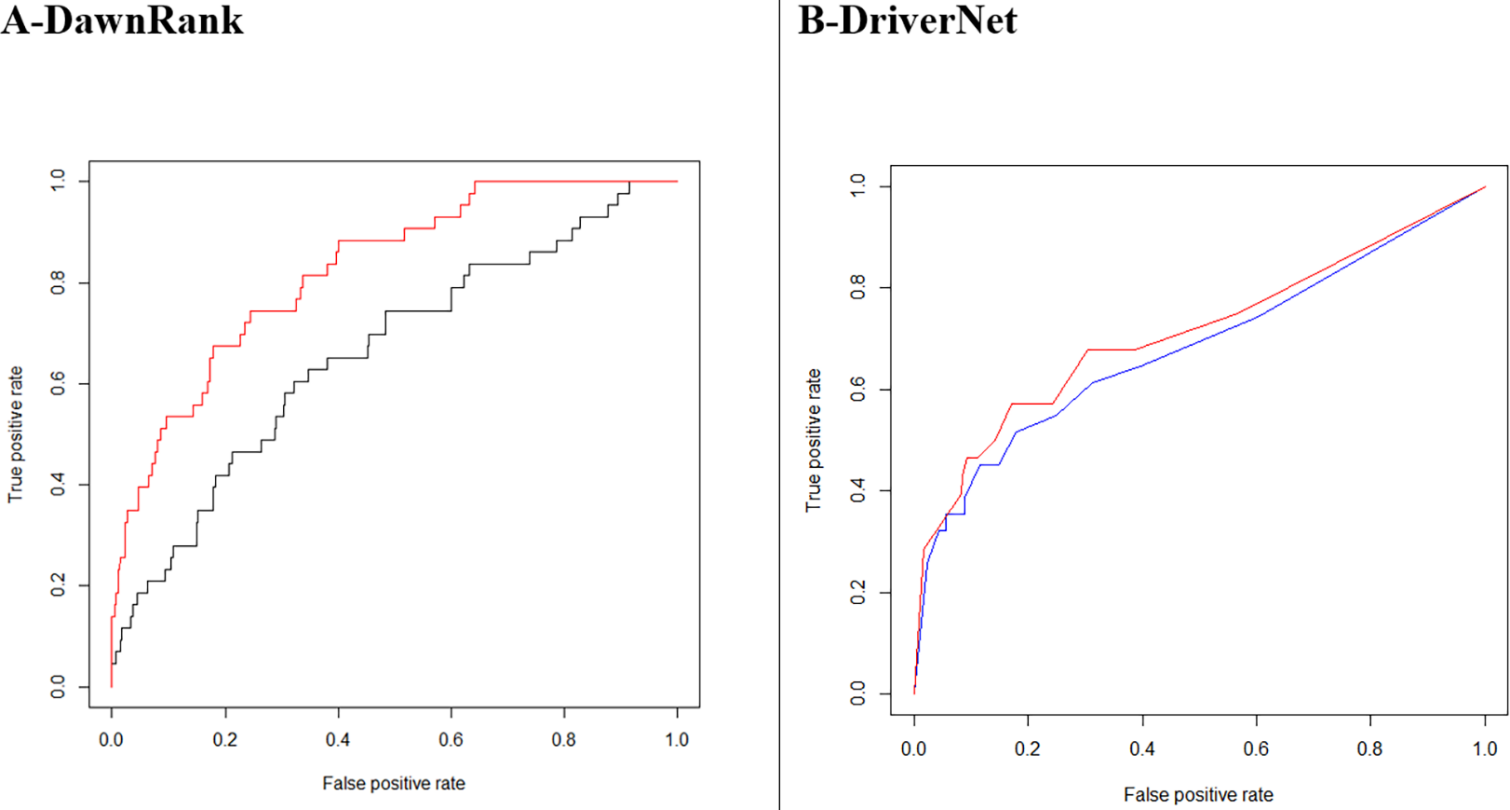
(A) ROC-DawnRank results (black) vs DawnRank results with weighted combined network (red). Area Under the Curve-AUC 0.6599 vs 0.8241. (B) ROC-DriverNet results with unweighted combined network (blue) vs DriverNet results with interactions above a cutoff weight of 0.17 (red). Area Under the Curve-AUC 0.6816 vs 0.7108.

DawnRank showed a larger improvement with its area under the ROC curve increasing from 0.6599 to 0.8241for breast cancer (Fig. 9A). A total of 235 driver genes were identified using GBM tumor samples with the DriverNet network compared to 308 for the combined network, an increase of 31%. The figures for cervical cancer were much higher, 337 and 1,201, respectively, an increase of more than 200%. These lists of genes are higher than Vogelstein’s list of 125, which used mutation characteristics to identify candidate oncogenes and tumor suppressor genes.

The potential of the weighted combined network to generate higher numbers of driver genes using the CGC list was also apparent, there being 7 more for GBM, and 102 more for cervical cancer. Three of these genes CBLC, CNOT3 and BMPR1A were common to both cancer types and were uniquely predicted using the combined network. When compared to Vogelstein’s list, 33 additional driver genes were predicted for cervical cancer using the weighted combined network. A total of 60 overlapping genes were predicted as driver genes across DawnRank breast cancer samples and Drivernet GBM samples, 20 of these were present in Vogelstein’s list. Of the remaining 40, we found seven to be identified by the CGC, the other 33 we have marked as candidate driver genes, requiring further study.

Our analysis also indicates that a larger network does not always produce better performance in the identification of cancer driver genes. A better quality network based on our weighting outperformed the unweighted network. In Fig. 9B, we see DriverNet produce better results when the low weighted interactions were removed. In the calculation of the weights assessing the linear bias correction given in Eq. (4), *α* and *β* took on values −0.07439 and 0.39795 respectively. We know each interaction weight is always greater than zero, so when summing positive values, the resultant weights are always greater than zero. The corrected combined weighting resulted in 18,541 interactions falling below the cut-off with a resultant network of 13,862 genes with 372,250 interactions.

## Conclusions

Gene interaction datasets have been constructed from databases such as KEGG, GO, NCBI, and Reactome. This is a limitation because the databases used are incomplete. The effectiveness of the use of interaction networks for the prediction of driver genes is heavily dependent on the quality of the gene interaction network. Our results confirm that the size and topological patterns of the interaction network directly impact the quality of the results generated by DriverNet and DawnRank. We found this increased the accuracy of the identification of driver genes by 17% and 28%, respectively. We have demonstrated the value of combining networks, which may be beneficial to other pathway-based methods. This network is also available to developers working on new gene interaction base solutions. Our approach of combining graphs and weighing their interactions can be used to improve other network graphs.

##  Supplemental Information

10.7717/peerj.2568/supp-1 Data S1 R data file, used by the R commands

10.7717/peerj.2568/supp-2 Data S2 Additional R data used by the R commands

10.7717/peerj.2568/supp-3 Supplemental Information 1 HTML file with R commands and Results

10.7717/peerj.2568/supp-4 Supplemental Information 2

## Additional Information and Declarations

### Data Availability

The following information was supplied regarding data availability: Public datasets were used and referenced in the manuscript. The raw data has been supplied as Supplementary Files.
