# Supplementary material for: The use of gene interaction networks to improve the identification of cancer driver genes
Source: PeerJ. 2017 Jan 26;5:e2568. doi: 10.7717/peerj.2568 (PMC5274523; doi:10.7717/peerj.2568)
Supplement: Supplemental Information 1 [file peerj-05-2568-s003.html]

Supplementary code and figures accompanying Combined gene interaction graphs improve identification of cancer driver genes


# Supplementary code and figures accompanying Combined gene interaction graphs improve identification of cancer driver genes

This document contains code used in our analysis. It is purely written in R using a series of R and Bioconductor packages. Copy the .Rdata files to your working directory, before executing this code. This report has been generated using the knitr R package (http://yihui.name/knitr/). For a complete list of packages and their versions please have a look at the end of this document. R version 3.2.5 with the following packages installed igraph, ROCR, caret, gplots and ggplot2

```
rm(list=ls()) 
load("InitVar1.rdata")
load("InitVar2.rdata")


rm(list=ls()) 
load("InitVar1.rdata")
load("InitVar2.rdata")
limit<-1


library(gplots)
```

```
## 
## Attaching package: 'gplots'
```

```
## The following object is masked from 'package:stats':
## 
##     lowess
```

```
library(igraph)
```

```
## 
## Attaching package: 'igraph'
```

```
## The following objects are masked from 'package:stats':
## 
##     decompose, spectrum
```

```
## The following object is masked from 'package:base':
## 
##     union
```

```
library(ROCR)
library(caret)
```

```
## Loading required package: lattice
```

```
## Loading required package: ggplot2
```

```
DRList<-data.frame(V(g1)$name)
VWList<-data.frame(V(G)$name)
DNList<-data.frame(V(gnet)$name)
colnames(DRList)<-c("id")
colnames(VWList)<-c("id")
colnames(DNList)<-c("id")
```

## Figure 8 -Gene and Interaction Overlap

```
v<-venn(list(DawnRank=DRList$id,VarWalker=VWList$id,DriverNet=DNList$id))
```

```
DRList<-data.frame(get.edgelist(g1, names=TRUE))
DRList$id <- paste(DRList$X1, DRList$X2, sep="-")
VWList<-data.frame(get.edgelist(G, names=TRUE))
VWList$id <- paste(VWList$X1, VWList$X2, sep="-")
DNList<-data.frame(get.edgelist(gnet, names=TRUE))
DNList$id <- paste(DNList$X1, DNList$X2, sep="-")

## Figure 8A -Interaction Overlap 
v<-venn(list(DawnRank=DRList$id,VarWalker=VWList$id,DriverNet=DNList$id))
```

```

```
# Construct weighted Network

X2 <- function(x){ y=sum(x)
          indx1=x>0
          x[indx1]=y-1
           return(x)
        }

GC1 <- GC[order(rownames(GC)),]
GC <- GC1[,order(colnames(GC1))]
Combine1<-apply(GC,2,X2)
max1<-max(Combine1)
Combine1<-Combine1/max1
##  Combine1 contains Combined Network

WeightedGC<- 0.5*Q + 0.5*Combine1
WeightedGC1<-WeightedGC
indx1<-WeightedGC1>0.17
WeightedGC1[indx1]=1
indx1<-WeightedGC1<=0.17
WeightedGC1[indx1]=0
## WeightedGC1 contains Weighted Combined network  after removing those with a low weight.
```

## Number of Interactions in Total Network vs Weighted Network

```
length(Combine1[Combine1>0])
```

```
## [1] 386755
```

```
length(WeightedGC1[WeightedGC1>0])
```

```
## [1] 372250
```

```
####### DawnRank ROC Analysis


# read in Vogelsteins List 
vogel<-vogelDrv
vogel$vval<-.6
limit<-1

# Function to accept a list of genes/scores, and assign those which occur in 
# Vogelsteins list as driver genes.  The prediction function format this data.
ROCInput1 <- function(values){
    vogel2<-merge(values, vogel, by = "gene", all.x = TRUE)
    vogel2[is.na(vogel2)] <- 0
        index<-vogel2$vval==.6
    vogel2$pred[index]<-1
    #library(ROCR)
    pred <- prediction( vogel2$pval, vogel2$pred)
    #perf2 <- performance(pred,"tpr","fpr")
    #perf2auc <- performance(pred,"auc")
    return(pred)
}


# Format the results using the RocInput1 function
# Calculate the TPR and FPR and AUC

pred<-ROCInput1(DRanksWC)
perfWC <- performance(pred,"tpr","fpr")
perfWCauc <- performance(pred,"auc")

pred<-ROCInput1(DRanks)
perf <- performance(pred,"tpr","fpr")
perfauc <- performance(pred,"auc")

#AUC DawnRank libary network and weighted combined network
perfauc@y.values
```

```
## [[1]]
## [1] 0.6599288
```

```
perfWCauc@y.values
```

```
## [[1]]
## [1] 0.8241376
```

## Figure 9A ROC- DawnRank results (black) vs DawnRank results with weighted combined network (red). Area Under the Curve -AUC 0.6599 vs 0.8241

```
plot(perf) 
lines(perfWC@x.values[[1]], perfWC@y.values[[1]], col = 2)
```

```
ROCInput2 <- function(values,genes,limit){
    values$pval1<-as.numeric(as.character(values$pval))
    values$pval1<-1-values$pval1
    gvalues<-values
    vogel2<-merge(gvalues, vogel, by = "gene", all.x = TRUE)
    vogel2[is.na(vogel2)] <- 0
    index<-vogel2$vval==.6
    vogel2$pred[index]<-1
    vogel2$vval1<-vogel2$vval+vogel2$pval1
    index<-vogel2$vval1==.6
    vogel2$pval1[index]<-limit
    pred <- prediction( vogel2$pval1, vogel2$pred)
    return(pred)
}

pval1<-pvalc
colnames(pval1)<-"pval"
pval1$gene<-rownames(pvalc)
pval1$pred<-0
gene<-data.frame(rownames(GC))
colnames(gene)<-"gene"
pred<-ROCInput2(pval1,gene,.0)
perfGC <- performance(pred,"tpr","fpr")
perfGCauc <- performance(pred,"auc")


pval1<-pvalwc
colnames(pval1)<-"pval"
pval1$gene<-rownames(pvalwc)
pval1$pred<-0
gene<-data.frame(rownames(WeightedGC1))
colnames(gene)<-"gene"
pred<-ROCInput2(pval1,gene,0)
```

```
## Warning in merge.data.frame(gvalues, vogel, by = "gene", all.x = TRUE):
## column name 'NA' is duplicated in the result
```

```
perfWGC <- performance(pred,"tpr","fpr")
perfWGCauc <- performance(pred,"auc")


perfGCauc@y.values
```

```
## [[1]]
## [1] 0.6815565
```

```
perfWGCauc@y.values
```

```
## [[1]]
## [1] 0.7108418
```

## Figure 9B ROC- DriverNet results with unweighted combined network (blue) vs DriverNet results with interactions above a cutoff weight of 0.17 (red). Area Under the Curve -AUC 0.6816 vs 0.7108

```
plot(perfGC,col = 4)
lines(perfWGC@x.values[[1]], perfWGC@y.values[[1]], col = 2)
```

```
sampleInfluenceGraph<-get.adjacency(gnet,sparse=FALSE)

pval1<-pval
colnames(pval1)<-"pval"
pval1$pval<-1
pval1$gene<-rownames(pval1)


genes<-data.frame(rownames(sampleInfluenceGraph))
colnames(genes)<-"gene"
gvalues<-merge(genes, pval1, by = "gene", all.x = TRUE)  
index<-is.na(gvalues)
gvalues[index]<-0.0
vogel<-vogelDrv
vogel$pred<-1
truth<-merge(genes, vogel, by = "gene", all.x = TRUE)
truth[is.na(truth)] <- 0
val<-as.factor(gvalues$pval)
pred<-as.factor(truth$pred)
cm<-confusionMatrix(val,pred)

pval1<-pvalwc
colnames(pval1)<-"pval"
pval1$pval<-1
pval1$gene<-rownames(pval1)

genes<-data.frame(rownames(WeightedGC1))
colnames(genes)<-"gene"
gvalues<-merge(genes, pval1, by = "gene", all.x = TRUE)  
index<-is.na(gvalues)
gvalues[index]<-0.0
vogel<-vogelDrv
vogel$pred<-1
truth<-merge(genes, vogel, by = "gene", all.x = TRUE)
truth[is.na(truth)] <- 0
val<-as.factor(gvalues$pval)
pred<-as.factor(truth$pred)
cmWeighted<-confusionMatrix(val,pred)
```

# Table 3 -Accuracy Standard Network vs Weighted Combined Network GBM

```
cm$overall['Accuracy']
```

```
##  Accuracy 
## 0.8159363
```

```
cmWeighted$overall['Accuracy']
```

```
##  Accuracy 
## 0.9733805
```

# The study identified 33 more candidate driver genes

```
vogel<-vogelDrv

v<-venn(list(Vogel=vogel$gene,Breast=DRanksWC$gene,Gbm=pvalwc$gene,Cervix=CervixWGc$gene))
```

```
isect <- attr(v, "intersection")
Candidate<-data.frame(isect$`Breast:Gbm:Cervix`)
colnames(Candidate)<-"gene"
c1<-data.frame(isect$`Breast:Gbm`)
colnames(c1)<-"gene"
Candidate<-rbind(Candidate,c1)


c1<-merge(Candidate, Census, by = "gene", all.x = TRUE)
index<-(!is.na(c1$Yes))

# Candidate genes not in Vogelstein's list but in CGC

Candidate<-data.frame(c1[index,1])
colnames(Candidate)<-"Candidate Driver Genes in CGC"

Candidate
```

```
##   Candidate Driver Genes in CGC
## 1                          CBLC
## 2                         CCND3
## 3                         CHEK2
## 4                         CSF3R
## 5                      HSP90AA1
## 6                          MDM2
## 7                           WAS
```

```
# New Candidates

index<-is.na(c1$Yes)
Candidate<-data.frame(c1[index,1])
colnames(Candidate)<-"New Candidate Driver Genes"
Candidate
```

```
##    New Candidate Driver Genes
## 1                       ACTN4
## 2                        AKT3
## 3                     CACNA1B
## 4                     CACNA1C
## 5                     COL11A1
## 6                      COL4A5
## 7                      COL6A2
## 8                      CTNNA3
## 9                         HGF
## 10                       INSR
## 11                      ITGB3
## 12                      KPNA2
## 13                       PAK3
## 14                    PIK3C2G
## 15                      PRKCG
## 16                      ROCK2
## 17                      ACSL4
## 18                     CHAF1A
## 19                      CNTN2
## 20                     COL1A2
## 21                      CSTF3
## 22                       FIGF
## 23                       GRM1
## 24                      HSPA8
## 25                       KNG1
## 26                       NOS3
## 27                     PIK3CG
## 28                     PPP3CA
## 29                      PRKDC
## 30                    RPS6KA3
## 31                      THBS1
## 32                        TNC
## 33                      WASF1
```

## Session

```
## R version 3.2.5 (2016-04-14)
## Platform: x86_64-w64-mingw32/x64 (64-bit)
## Running under: Windows 8.1 x64 (build 9600)
## 
## locale:
## [1] LC_COLLATE=English_United States.1252 
## [2] LC_CTYPE=English_United States.1252   
## [3] LC_MONETARY=English_United States.1252
## [4] LC_NUMERIC=C                          
## [5] LC_TIME=English_United States.1252    
## 
## attached base packages:
## [1] stats     graphics  grDevices utils     datasets  methods   base     
## 
## other attached packages:
## [1] caret_6.0-70    ggplot2_2.1.0   lattice_0.20-33 ROCR_1.0-7     
## [5] igraph_1.0.1    gplots_3.0.1   
## 
## loaded via a namespace (and not attached):
##  [1] Rcpp_0.12.4        nloptr_1.0.4       formatR_1.4       
##  [4] plyr_1.8.4         class_7.3-14       bitops_1.0-6      
##  [7] iterators_1.0.8    tools_3.2.5        digest_0.6.9      
## [10] lme4_1.1-12        evaluate_0.9       gtable_0.2.0      
## [13] nlme_3.1-127       mgcv_1.8-12        Matrix_1.2-5      
## [16] foreach_1.4.3      parallel_3.2.5     yaml_2.1.13       
## [19] SparseM_1.7        e1071_1.6-7        stringr_1.0.0     
## [22] knitr_1.13         MatrixModels_0.4-1 gtools_3.5.0      
## [25] caTools_1.17.1     stats4_3.2.5       grid_3.2.5        
## [28] nnet_7.3-12        rmarkdown_0.9.6    gdata_2.17.0      
## [31] minqa_1.2.4        reshape2_1.4.1     car_2.1-2         
## [34] magrittr_1.5       scales_0.4.0       codetools_0.2-14  
## [37] htmltools_0.3.5    MASS_7.3-45        splines_3.2.5     
## [40] pbkrtest_0.4-6     colorspace_1.2-6   quantreg_5.24     
## [43] KernSmooth_2.23-15 stringi_1.1.1      munsell_0.4.3
```
