# Supplementary figures and images for: The use of gene interaction networks to improve the identification of cancer driver genes
Source: PeerJ. 2017 Jan 26;5:e2568. doi: 10.7717/peerj.2568 (PMC5274523; doi:10.7717/peerj.2568)

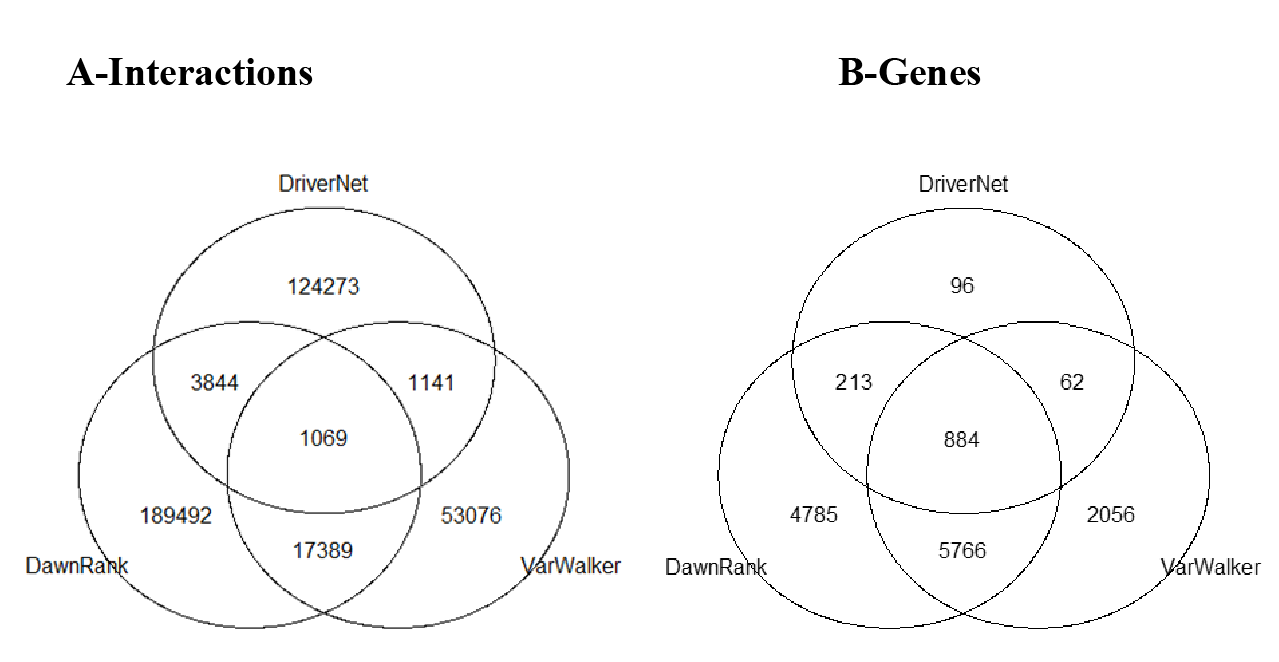

Supplement: Supplemental Information 2 [file peerj-05-2568-s004.png]
